# Four-Stage Multi-Physics Simulations to Assist Temperature Sensor Design for Industrial-Scale Coal-Fired Boiler

**DOI:** 10.3390/s24010154

**Published:** 2023-12-27

**Authors:** Tanuj Gupta, Mahabubur Rahman, Xinyu Jiao, Yongji Wu, Chethan K. Acharya, Dock R. Houston, Susan Maley, Junhang Dong, Hai Xiao, Huijuan Zhao

**Affiliations:** 1 Department of Mechanical Engineering, Clemson University, Clemson, SC 29634, USA; tgupta@clemson.edu (T.G.); mahabur@clemson.edu (M.R.); 2 Department of Electrical and Computer Engineering, Clemson University, Clemson, SC 29634, USAyongjiw@clemson.edu (Y.W.);; 3 Southern Company, Birmingham, AL 35203, USA; 4 Machining and Technical Services, Clemson University, Clemson, SC 29634, USA; dock423@clemson.edu; 5 Electric Power Research Institute, Charlotte, NC 28262, USA; 6 College of Engineering and Applied Science, University of Cincinnati, Cincinnati, OH 45221, USA

**Keywords:** computational fluid dynamics, heat transfer, structural mechanics, multi-physics modeling, boiler tube, sensor design

## Abstract

The growth of renewable energy sources presents a pressing challenge to the operation and maintenance of existing fossil fuel power plants, given that fossil fuel remains the predominant fuel source, responsible for over 60% of electricity generation in the United States. One of the main concerns within these fossil fuel power plants is the unpredictable failure of boiler tubes, resulting in emergency maintenance with significant economic and societal consequences. A reliable high-temperature sensor is necessary for in situ monitoring of boiler tubes and the safety of fossil fuel power plants. In this study, a comprehensive four-stage multi-physics computational framework is developed to assist the design, optimization installation, and operation of the high-temperature stainless-steel and quartz coaxial cable sensor (SSQ-CCS) for coal-fired boiler applications. With the consideration of various operation conditions, we predict the distributions of flue gas temperatures within coal-fired boilers, the temperature correlation between the boiler tube and SSQ-CCS, and the safety of SSQ-CCS. With the simulation-guided sensor installation plan, the newly designed SSQ-CCSs have been employed for field testing for more than 430 days. The computational framework developed in this work can guide the future operation of coal-fired plants and other power plants for the safety prediction of boiler operations.

## 1. Introduction

Even though the clean energy revolution has been rapidly expanded in recent years due to its strong environmental sustainability and tremendous economic opportunity, over 60% of the electricity generated is still from fossil fuels (such as natural gas, coal, petroleum, etc.) in the U.S., as reported in 2023 [1]. One of the major risks in coal-fired and gas-fired power plant management is unexpected boiler tube failures, accounting for over 60% of unplanned power plant outages [2,3,4]. The boiler tubes work as high-temperature heat exchangers between steam flow and flue gas in industrial-scale coal-fired boilers, dealing with harsh operating conditions such as overheating, waterside/fireside corrosion, fly ash erosion, and ash deposition, summarized in a comprehensive review on coal-fired steam power plants [5]. Meanwhile, the increasing contributions of renewable energy sources require fossil fuel power plants to manage frequent load exchanges, increased start-ups/shutdowns, and longer layups for economic reasons [6,7,8], making the maintenance schedules insufficient to keep up with the reliability requirements. Such frequent load exchange can lead to unexpected high temperatures and insufficient heat transfer on boiler tubes, which would degenerate the mechanical and thermal properties of the tube materials, such as their heat conductivity, mechanical strength, and creep life [9,10]. As temperature is the dominating factor in material property degeneration and boiler tube failure, it is important to monitor the temperature distribution of the boiler tube to better predict the boiler tube service life and plan for boiler maintenance and outage in a more economical way.

Existing harsh environment sensors are mostly point sensors to acquire information at a specific location. While rigorously packaged thermocouples and resistance temperature detectors (RTDs) have been used to measure the temperatures of superheaters, reheaters, headers, economizers, and wall tubes, not only is their installation cumbersome due to the wiring but also they quickly become very costly if multiple locations need to be monitored. Optical sensors have been widely adopted under high-temperature environments [11,12,13]. However, the installation of optical sensors is difficult due to their fragile material properties in nature. There is always a need to design a low-cost and robust distributive high-temperature sensor with easy installation and reliable operation in harsh environments. 

To facilitate the sensor design and optimization, computational modeling and simulation of the boiler performance are facing multiple challenges. The system involves complex physics, such as multiphase flow, combustion, heat transfer, and mechanical loadings. A wide range of length scales needs to be considered, from the boiler structure (in the meter scale) to the boiler tube thickness (in the sub-millimeter scale). Most of the established simulations simplify the steam tube panels as thin plates with fixed thermal boundary conditions to ease the computational cost [14,15,16,17,18,19,20,21,22,23,24]. Chen et al. considered the pendant superheater to be thin panels. The whole section of the final superheater and the final reheater section were modeled as two simplified control volumes [14]. Laubscher et al. evaluated the temperature distribution in the superheater region for varying loads, considering the steam tubes to be flat panels [15]. Schuhbauer et al. considered the heat exchangers as porous media panels consisting of a heat sink to simulate heat taken away by steam flow [19]. Yu et al. assumed all stages of superheaters as flat surfaces [20]. Modlinski et al. developed a method for predicting the temperature of metal plates in pendant reheaters by coupling a 3D boiler model with a 1D heated pipe flow model [21]. While these simplifications in steam panel modeling significantly reduce the computational cost, they are unable to accurately predict temperature variations along the tubes and across different sections of the tubes. Akkinepally et al. performed multiscale modeling of a boiler and a single tube section [22]. They considered a single bundle of eighteen reheater tube sections and investigated the boiler tube temperature for two different inflows of flue gas velocity. They found that the higher velocity of flue gas led to higher boiler tube temperature. Granda et al. considered a single row of second-stage superheaters to perform the steady-state analysis and the transient-state analysis of the temperature distribution in the tubes [23]. Qi et al. evaluated the tube wall temperature by combining a 3D boiler model with a detailed tube arrangement of a superheater section and investigated the effect of the oxide scale on the tube wall temperature [24]. During the boiler operation, monitoring stress distribution and variation in boiler tubes is essential for detecting potential tube failure. Zhang et al. studied the heat transfer and static performance of the vertical water wall tubes of coal-fired boilers using one-way fluid–structure interactions (FSI) [25]. Botha et al. adopted the one-way FSI method to calculate the stress field of boiler tubes [26]. Madejski et al. performed a thermomechanical analysis of the steam tubes of the second stage of the platen superheater [27]. In summary, it is necessary to establish a robust integration of the multi-physics modeling framework to systematically simulate the full-scale coal-fired boiler system, both integrating the broad length scale of the complex system and monitoring the health condition of the boiler tubes by accurately predicting their temperature and stress distributions. 

To enhance the monitoring of the distributed temperature of boiler tubes, we have developed a cost-effective stainless-steel and quartz coaxial cable sensor (SSQ-CCS) for in situ distributed temperature monitoring in high-temperature harsh environments [28]. Through laboratory-scale testing, we demonstrated that the first-of-the-kind SSQ-CCS maintains accurate temperature measurements with a resolution of 0.5 °C at 600 °C for over 350 h [28]. We have installed the SSQ-CCSs for field testing with easy accessibility. The field test results confirmed the accuracy of SSQ-CCS temperature measurement when compared with the thermocouple data [28]. We have proved the feasibility of adopting SSQ-CCSs for temperature monitoring in harsh environments such as coal-fired boiler tubes. 

In support of the design, optimization, installation, and evaluation of SSQ-CCSs [28], this work proposes and establishes a four-stage multi-physics simulation framework through commercial modeling software ANSYS^®^ 2022 R2 [29] to consider the broad length scale of the complex coal-fired boiler system by dividing it into four distinct length scales. Each scale maintains its rigorous geometry specifications for accurate predictions of the boiler tube temperature and stress distributions. Various operating conditions are investigated to ensure the reliable performance of the SSQ-CCSs. The temperature correlation between the steam, tube, and SSQ-CCS is established. The proposed four-stage multi-physics simulation framework is proven to precisely predict the temperature variation in the tubes and validate the performance of the SSQ-CCS. In the following, Section 2 briefly introduces the four-stage multi-physics simulation framework and setup of the simulation models; Section 3 presents the results and discussion of (1) the sensor installation locations [30], (2) the steam and tube temperature variations under different conditions, (3) the tube and sensor temperature correlations and stress variations with respect to different conditions, and (4) the SSQ-CCS assembly and field testing. The conclusion and future perspectives are presented in the last section. 

## 2. Methodology and Simulation Setup

### 2.1. Four-Stage Multi-Physics Framework

**Figure 1:**
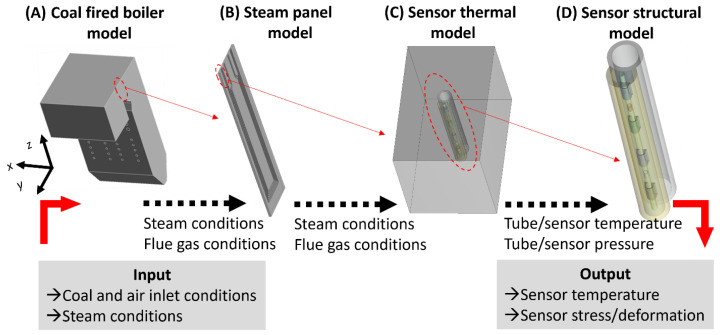
Schematic of the four-stage multi-physics modeling framework.

Due to the significant length-scale difference from an industrial-scale coal-fired boiler (order of 10 m) to the thickness of the coaxial cable (order of 0.1 mm), it is impractical to simulate the sensor performance within the full-scale 3D coal-fired boiler. We define the multi-physics framework in four stages to conduct the simulations at different length scales, listed in Figure 1. This framework includes the direct coupling of computational fluid dynamics (CFD) and heat transfer (HT) analysis and the indirect coupling to the FSI and structural-thermal (ST) analysis. The objectives of the four stages and the corresponding analysis tools are summarized as follows: (A) The real-size coal-fired boiler model (CFD, HT): the temperature and velocity distributions of flue gas within the boiler region under various conditions are predicted, aiming to establish and evaluate the SSQ-CCS installation criteria. (B) The real-size steam panel model (CFD, HT): the temperature distributions of the steam and tubes are investigated with respect to various operation parameters, aiming to identify the dominant control parameters to the temperature field of the steam and boiler tubes. (C) The SSQ-CCS thermal model (CFD, HT): the temperature field on the SSQ-CCS is predicted based on the input steam and flue gas conditions from the stage (B) model. (D) The SSQ-CCS structural model (ST): the stress distribution and deformation of the SSQ-CCS are evaluated with respect to the operation safety of the SSQ-CCS. The thick red arrows represent the inputs and outputs of the four-stage multi-physics modeling framework. The black dash arrows represent the data exchange and flow. The dashed oval with a thin red arrow reflects the relation between the models and the length-scale reduction in the simulation domain. Such a framework and data flow enable the modeling capabilities of accurate predictions of the SSQ-CCS performance in the industrial-scale coal-fired boiler with respect to various conditions. Such a modeling framework and capability can efficiently and effectively assist the sensor design process. 

### 2.2. Multi-Physics Coupling and Model Setup

In the stage (A) model, the directly coupled CFD and HT analysis is employed through the classical finite volume method in ANSYS Fluent with the considerations of the chemical reaction, turbulence, tracking of combustibles, and radiative heat transfer [15,20,29,30]. Due to the consideration of coal-fire reaction, the energy conservation equation is defined as follows:

(1) $$\frac{\partial }{\partial t}\left(\rho E\right)+\nabla \cdot \left(\vec{u}\left(\rho E+p\right)\right)=\nabla \cdot \left(k_{eff}\nabla T-\sum_{i}h_{i}{\vec{J}}_{i}+\left({\tilde{\tau }}_{eff}\cdot \vec{u}\right)\right)+S_{h}$$

where *t* is time, *ρ* is density, *E* is the total energy for the steady incompressible flow, $\vec{u}$ is the flow velocity, *p* is the pressure, $k_{eff}$ is the effective conductivity due to turbulence and dissipation, T is the temperature, $h_{i}$ and ${\vec{J}}_{i}$ are the enthalpy and diffusion flux of the species, respectively. ${\tilde{\tau }}_{eff}$ is the effective stress tensor due to the fluid viscosity, and $S_{h}$ is the energy released due to the gas phase reaction, which is defined by the Eddy dissipation model [31]. The coal particles are defined by a discrete phase model. The setup of both the Eddy dissipation model and the discrete phase model can be found in Ref. [30]. The Discrete Ordinate model is adopted to account for the radiative heat transfer [32]. The semi-implicit method for the pressure-linked equations (SIMPLE) algorithm is used for pressure-velocity coupling [33]. 

In the stage (B) model and the stage (C) model, the $S_{h}$ term in the energy conservation equation is not considered, as the flue gas temperature profile has been obtained through the stage (A) model. We impose the temperature and pressure fields from the stage (C) model to the stage (D) model to calculate the stress and deformation field of the tube and sensor. Considering the thermal expansion, the stress-strain relation is defined as follows:

(2) $$\vec{\sigma }=D\left(\vec{\varepsilon }-\vec{\alpha }\cdot \Delta T\right)$$

where $\vec{\sigma }$ and $\vec{\varepsilon }$ are the stress and strain vectors, **D** is the elastic stiffness matrix, $\vec{\alpha }$ is the thermal expansion coefficient vector, and ΔT is the temperature difference with respect to the initial configuration (room temperature).

**Figure 2:**
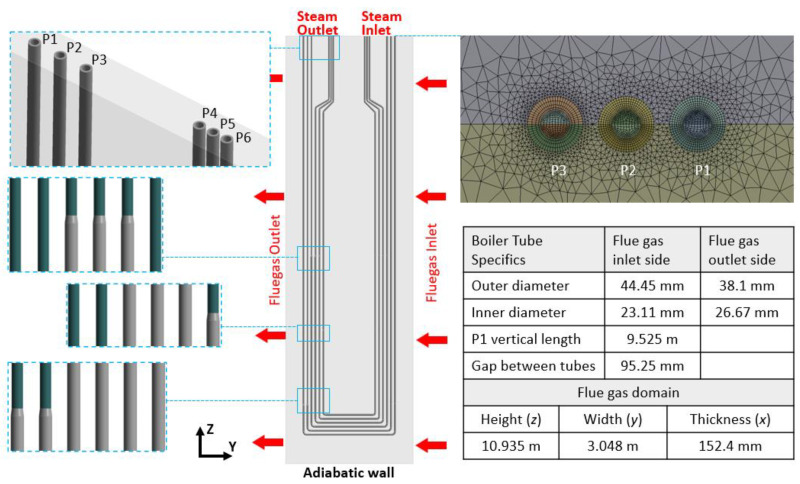
Geometry, boundary conditions, and representative mesh of the stage (B) steam panel model.

The stage (A) model with the corresponding model parameters is described in our previous publication [30]. The stage (B) steam panel model consists of one panel with six tubes, the flue gas domain, and the steam domain, shown in Figure 2. The geometry information of the steam panel is referenced from the on-site SSQ-CCS testing facility. Major geometry specifications are listed in the embedded table. The tubes (P1–P6) are made of stainless-steel SS-347-H with two different cross-sections. It is designed to be thicker on the flue gas inlet side than on the flue gas outlet side. The zoom-in figures show the transition of the tube cross-section. The thermal conductivity, specific heat, and density of SS-347-H are defined as 21.4 W/(m·K), 500 J/(kg·K), and 8030 kg/m^3^, respectively. The simulation domain boundaries along the *x* and *z* directions are simplified to be adiabatic walls except for the steam inlets and outlets. The flue gas inlet and outlet are marked in the figure. Both the steam and flue gas flow are defined to be turbulent with no-slip conditions on the wall. This stage (B) model has 13.3 million hexahedral-type elements for both the steam tube domain and the steam domain. There are 38.62 million tetrahedral elements in the flue gas domain. The mesh near the steam inlet of pipe 1 to pipe 3 is presented in Figure 2. A dense mesh is generated near the steam tube/flue gas and steam tube/steam interfaces to ensure the simulation accuracy, and a rough mesh is used in the remaining flue gas domain to ensure the simulation efficiency. 

**Figure 3:**
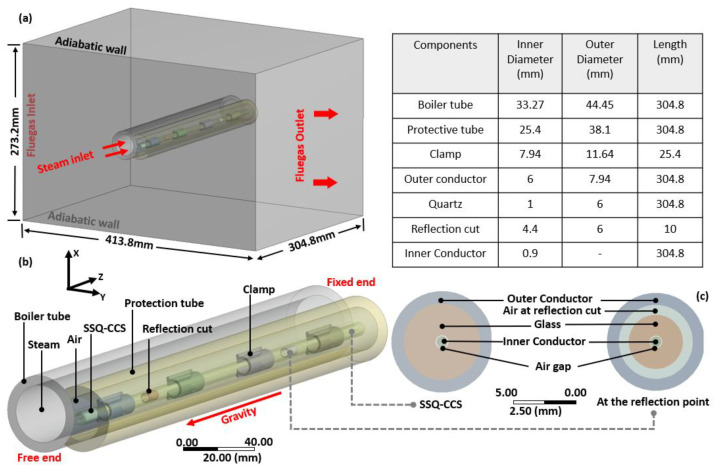
Geometry and boundary conditions of the stage (C) and (D) SSQ-CCS models. (**a**) the simulation domain of stage (C) model with dimensions and main boundary conditions. (**b**) the simulation domain of stage (D) model. (**c**) the cross-section of the SSQ-CCS in stage (D) model at the location with the reflection cut (on the right) and without the reflection cut (on the left).

The stage (C) and (D) models are presented in Figure 3a and Figure 3b, respectively. The design, fabrication, and temperature-sensing mechanism of the SSQ-CSS has been reported in our previous publication [28]. The SSQ-CCS includes an outer conductor and an inner conductor made of SS-347-H, the same as the tube. In between the two conductors is a glass tube, acting as the medium for signal transmission. Hoop-shaped reflection cuts on the glass tube serve as signal reflectors. The cross-section views of the SSQ-CCS with/without the reflection cuts are shown in Figure 3c. The distance between the cuts impacts the conversion between the sensing signal and measured temperature [28]. The SSQ-CCS is attached to the boiler tube by welded clamps. A crescent-shaped protection tube is welded to the tube to prevent direct contact between the SSQ-CCS and the flue gas (Figure 3b). Air fills the rest of the domains embraced by the protection tube. The geometry properties are given in the embedded table in Figure 3. The material properties of SS-347-H, glass, and air are listed in Table 1. The thermal conductivities of SS-347-H and air are defined as temperature-dependent for better accuracy [34,35], as they impact the calculation of the structure mechanics in the stage (D) model. The welded interfaces are perfectly bonded. The other contacts are considered frictionless. The contact areas are modeled with a trim tolerance of 0.8 mm. The boundary conditions are listed in Figure 3. Since the boiler tube assembly is fixed at the steam outlet, the tube at the steam outlet end is considered fixed in the stage (D) model. There are more than 15 million elements in the flue gas domain in the stage (C) model and 2.5 million hexagonal elements in the stage (D) model. Such mesh ensures the simulation convergence and accuracy. 

## 3. Results and Discussion

### 3.1. Prediction of Sensor Installation Location

**Figure 4:**
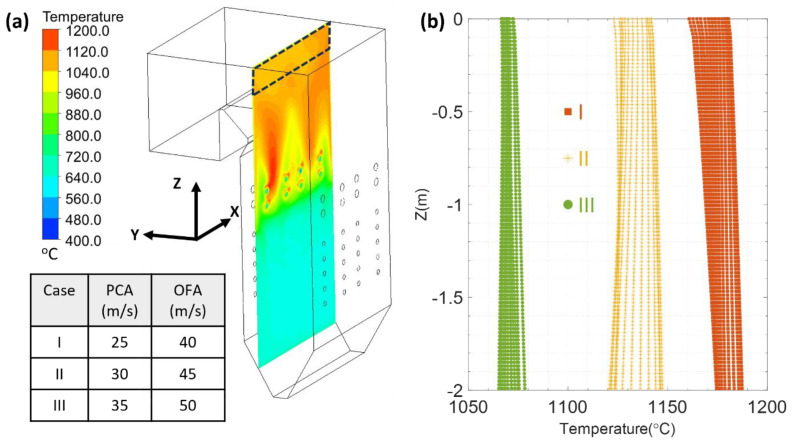
(**a**) Flue gas temperature profile at a vertical section of the boiler; (**b**) flue gas temperature variation at the selected location with respect to various PCA and OFA velocity conditions. The PCA and OFA temperatures are set to be 85 °C and 323 °C, respectively. No simplified steam panel is considered [29].

To ensure the success of the SSQ-CCS testing and calibration, the preferred installation location in the boiler is where the flue gas temperature distribution is stable and steady. Throughout the stage (A) model, we have analyzed the temperature distribution near the steam panels and proposed the SSQ-CCS installation criteria: it should be installed (1) away from the side walls, (2) closer to the penthouse of the boiler, and (3) away from the direct impact of the flue gas [30]. As shown in Figure 4a, the region marked with a black dashed line has the minimum temperature variation and can be considered an ideal location for SSQ-CCS installation. We also investigated three coal and air conditions with respect to different primary coal and air (PCA) velocities and overfire air (OFA) velocities (embedded Table in Figure 4). The temperature variation in the flue gas in the black dashed-line box is plotted in Figure 4b. Within a 2 m range from the penthouse of the boiler, the temperature fluctuation is less than 30 °C and 2.6% of the flue gas temperature. This SSQ-CCS installation criteria has been adopted in the field testing reported in Ref. [28].

### 3.2. Prediction of Steam Tube Temperature Variation

**Figure 5:**
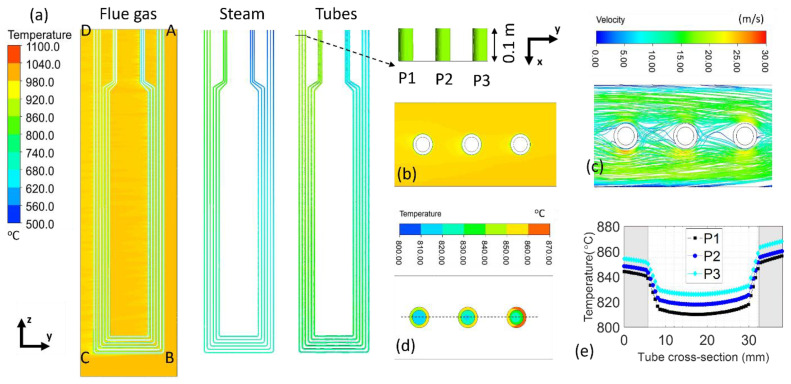
(**a**) Temperature profile of the flue gas, steam, and outer wall of the tubes in the y-z view. The input parameters of the presented case study are the flue gas velocity of 14.5 m/s, flue gas temperature of 1000 °C, steam temperature of 540 °C, and steam mass flow rate of 0.1 kg/s. (**b**–**d**) The x-y view of the flue gas temperature, flue gas velocity, and steam/tube temperature near tubes P1–P3 at z = 0.2 m below the top boundary. (**e**) The temperature variation in steam/tube along the tube diameter. The gray blocks reflect the tube domain, and the white region in the middle reflects the steam domain.

To understand the temperature distributions of the tubes in the steam panel as well as the temperature variations with respect to different input parameters, we adopt the stage (B) model to perform the parametric study. The stage (B) model includes six tubes in one panel, with controlled input parameters such as flue gas temperature and velocity, inlet steam temperature, and inlet steam mass flow rate. Figure 5 presents the temperature profile of the flue gas, steam, and tubes of one case study. The steam temperature increases while passing through the boiler tubes, heated up by the surrounding flue gas. At the cross-section, 0.1 m below the steam outlets of tubes P1, P2, andP3, Figure 5b,c presents the temperature and velocity profile of the flue gas; Figure 5d presents the temperature profile of the steam and tubes. The flue gas temperature is steady. The tube temperature shows a decreasing trend away from the direction of the incoming flue gas. The temperature distribution of each tube varies as well. The steam temperature is lower than the tube temperature. We plot the steam/tube temperature along the diameters of P1, P2, andP3 at 0.1 m below the steam outlets and present it in Figure 5d. The steam has a much lower temperature at the center of the tube than that near the inner wall of the tube. Moreover, tube P3 has a higher cross-section temperature than tube P1 because it is closer to the flue gas inlet. The temperature difference along the thickness of the tube is below 5 °C. The temperature difference along the cross-sections of the tubes is around 16 ± 0.4 °C, less than 2% of the tube temperature. Such small temperature differences along the cross-section can be neglected when establishing the temperature correlations between the tube and the SSQ-CCS in the following studies.

**Figure 6:**
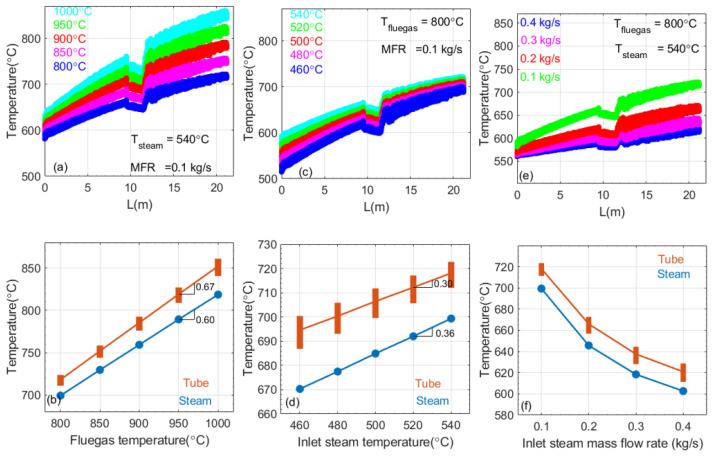
Parametric study of tube P1’s temperature variation with respect to (**a**) flue gas temperature, (**c**) steam temperature, and (**e**) steam mass flow rate. The corresponding comparison of the tube temperature at 0.1 m from the stem outlet and steam temperature at the steam outlet is plotted in (**b**), (**d**), and (**f**), respectively. The circular symbol reflects the steam temperature at the outlet. The rectangular symbol reflects the tube temperature at 0.1 m to the steam outlet. The symbol length reflects the temperature fluctuation on the cross-section of the tube.

Since the SSQ-CCS is installed at tube P1, nearly 0.1 m from the steam outlet, we will focus on tube P1’s temperature variation with respect to various input parameters. Figure 6a,c,e presents tube P1’s temperature variation along the steam flow direction (A→B→C→D in Figure 5a) with respect to (a) the flue gas temperature (T_flue gas_) variation, (c) the steam temperature (T_steam_) variation, and (e) the steam mass flow rate (MFR) variation. The flue gas velocity remains at 14.5 m/s in this study. The tube temperature presents an increasing trend when the tube is perpendicular to the flue gas direction. As the steam temperature increases while traveling through the tube, less heat will be carried away by direct heat conduction from the steam, tube, and flue gas. Increasing the flue gas temperature leads to an increase in the tube temperature because more heat is applied to the steam panel. Increasing the steam temperature leads to an increase in the tube temperature because less heat will be carried away. The increased steam mass flow rate will reduce the tube temperature as the low-speed steam will take more heat away from a such process. As shown in Figure 5e, there is a temperature variation along the cross-section of the tube. In Figure 6a,c,e, the thickness of the plot reflects such temperature variation at any cross-section along tube P1. It reaches the maximum value near turning point C in Figure 5a. Since the steam outlet temperature is monitored with the temperature sensor installed at the penthouse of the boiler, we further compared the temperature at 0.1 m on tube P1 near the steam outlet to the steam temperature at the outlet with respect to the above-mentioned parameter variations, shown in Figure 6b,d,f. The outlet steam and tube temperature present a linear increasing trend with respect to the flue gas temperature and inlet steam temperature, respectively. However, the gradients are different, as shown in Figure 6b,d. The tube temperature variation is more sensitive to the flue gas temperature than the inlet steam temperature. Meanwhile, the temperature of outlet steam and tube present a nonlinear decreasing trend with respect to the inlet steam MFR. Increasing the MFR of the inlet steam would significantly reduce the tube temperature. The correlation between the outlet steam temperature and tube temperature is consistent. Such a parametric study provides us guidance for future in situ tube temperature monitoring and adjustment by controlling these input parameters. 

### 3.3. Prediction of SSQ-CCS Performance

**Figure 7:**
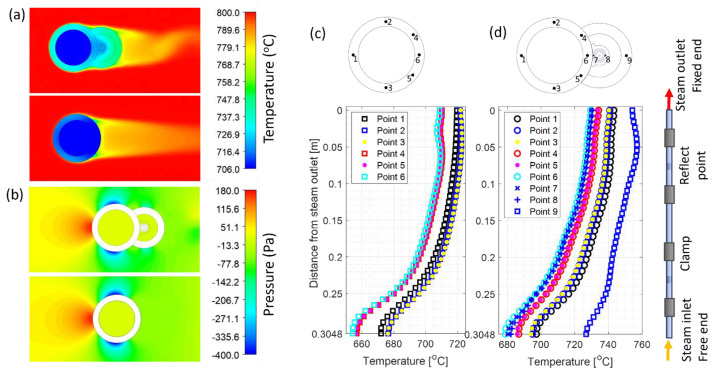
(**a**) The temperature and (**b**) pressure difference with/without sensor installation. (**c**) The temperature distribution near the steam outlet without sensor installation. (**d**) The temperature distribution near the steam outlet with sensor installation.

When the SSQ-CCS and protection tube are welded to the boiler tube, the temperature distribution on the boiler tube is influenced by the attachment, especially where the sensor is welded. To understand the impact on the temperature distribution of the boiler tube due to the installation of the SSQ-CCS sensor, we performed a case study by adopting the stage (C) model to predict the temperature distribution of the boiler tube with and without the sensor installation. In this case study, T_steam_ = 440 °C, T_flue gas_ = 800 °C, the flue gas velocity is 14.5 m/s, the flue gas pressure is set to be 1 atm, and the steam MFR = 0.1 kg/s. The contour plots of the temperature and pressure distribution at the steam outlet cross-section are shown in Figure 7a,b. Since the sensor and protection tube are installed next to the flue gas outlet direction, their impact on the pressure profile of the flue gas near the boiler tube is limited, as shown in Figure 7b. However, the temperature distribution on the boiler tube is significantly impacted. Figure 7c,d presents the temperature distribution on the boiler tube along the axial direction at the selected locations without and with the sensor/protection tube installed. Point 1 is directly facing the incoming flue gas. Point 6 is against the incoming flue gas direction and directly contacts the sensor if installed. The vertical axis of the plot is the distance from the steam outlet. The steam inlet and outlet are at 0.3048 m and 0 m on the vertical axis, respectively. The heat transfer is fully developed near the steam outlet, and a stable temperature distribution on the boiler tube can be observed. Without the sensor attachment, point 2 (3) has a slightly higher temperature than point 1. With the sensor attachment, point 1 has a higher temperature compared to points 2 (3), 4 (5), and 6. Points 7 and 8 are on the outer conductor of the sensor, presenting a similar temperature to point 6. On the protection tube, point 9 presents the highest temperature of the cross-section. Overall, the temperature rises around 10 °C due to the attached sensor and protection tube. 

**Figure 8:**
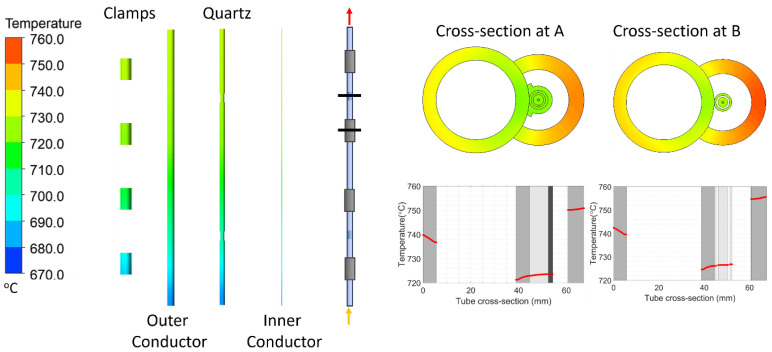
Temperature profile of the sensor along the axis direction and the cross-section view at points A and B. Point A is at the reflection cut, and point B is at the clamp. The temperature distribution along the tube cross-section is also plotted. The gray shades represent the boiler tube, sensor, clamp, and protection tube correspondingly.

The output of the stage (C) model includes the pressure and temperature distribution profile of the boiler tube, sensor, and protection tube. Under the same case study, the temperature profile on the parts of the sensor, as well as the temperature contour at cross-sections A and B, are presented in Figure 8. The temperature distribution along the symmetric axis of cross-sections A and B are plotted as well. The temperature difference along the cross-section B (0.1 m from the steam outlet) is 19 °C. Due to the sensor attachment, the temperature variation across the thickness near the sensor is much smaller than that near the flue gas side.

**Figure 9:**
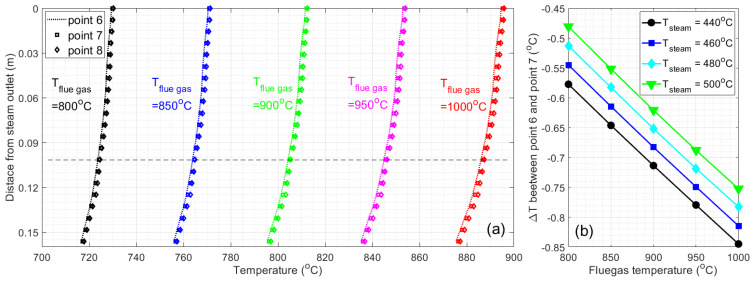
(**a**) Temperature variation/compression between the outer boiler tube and outer conductor of the sensor at different flue gas temperatures. (**b**) Temperature difference between point 6 and point 7 at different flue gas temperatures and steam temperatures.

To define the correlation between the sensor temperature and boiler tube temperature, we systematically investigate the temperature variation in the boiler tube and SSQ-CCS within the [0, 0.15 m] range near the steam outlet with respect to the variation in the steam temperature and flue gas temperature. Figure 9a presents the temperature variation in points 6, 7, and 8 with T_steam_ = 440 °C and T_flue gas_ varying from 800 °C to 1000 °C. The other input parameters are identical to the set up in the aforementioned case study. The difference between the sensor temperature (points 7 and 8) and the boiler tube temperature (point 6) is negligible with respect to flue gas temperature variation. We select the location of 0.1 m below the steam outlet (as the dashed line shown in Figure 9a) and plot the temperature difference between point 6 (boiler tube) and point 7 (outer conductor) with respect to the steam temperature variation and flue gas temperature variation, shown in Figure 9b. The overall temperature difference is within 1 °C. Such a temperature difference increases with respect to the steam temperature increase but decreases with respect to the flue gas temperature increase. 

**Figure 10:**
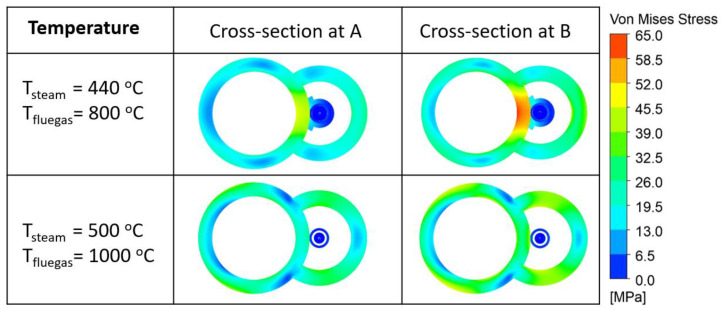
The von Mises stress distribution on cross-sections A and B defined in Figure 8 at different steam/flue gas temperature setups.

Stress under elevated temperatures is one of the main reasons for the material degradation of the boiler tube. To ensure the liability of the SSQ-CCS sensor, monitoring the stress distribution and magnitude is necessary. In the stage (D) model, we adopted the structural thermal (ST) module in ANSYS to predict the stress and deformation of the sensor. The input temperature profile and pressure distribution on the surface of the boiler tube/sensor are provided through the output of the stage (C) model. Figure 10 presents the von Mises stress distribution at cross-sections A and B (Figure 8) with respect to two steam/flue gas temperature conditions. The stress on the sensor is negligible, especially along the axial direction. There is a stress concentration at the tube/sensor interface at cross-section B, where the clamp is welded to the boiler tube in order to ensure contact between the boiler tube and sensor due to the welding of the clamps on the bolder tube. However, the stress magnitude (65 MPa) is much less than the yielding strength of SS-347-H (205 MPa).

### 3.4. SSQ-CCS Assembly and Testing

**Figure 11:**
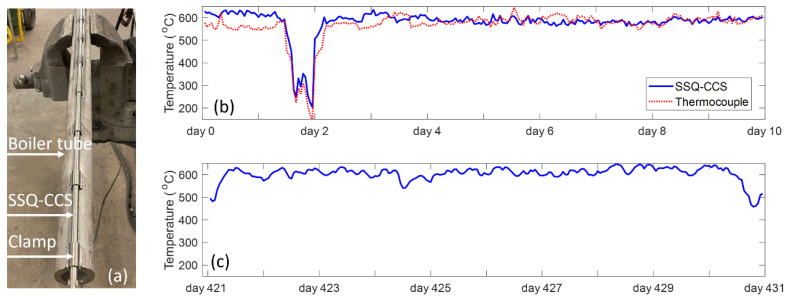
(**a**) The assembly of the SSQ-CCS and steam tube; (**b**) SSQ-CCS testing data from the first ten days of operation; (**c**) SSQ-CCS testing data from day 421 to day 431 of operation.

In this work, we have fabricated the designed SSQ-CCSs for on-site field-testing purposes. Our previous work [28] systematically detailed the sensor design, fabrication, installation, and testing procedures. Figure 11a displays a photograph of the sensor-tube assembly. The SSQ-CCS is attached to the boiler tube through a series of welded clamps. The protection tube is not shown in this photograph. For field testing, our sensors are installed together with a set of thermocouples for data validation purposes. To date, our SSQ-CCSs have continuously monitored the boiler tube temperature for more than 430 days, while the installed thermocouples stopped working within a year. Figure 11b presents the first ten-day temperature data from both the SSQ-CCS and thermocouple. The observed normal operating temperature at the boiler tube is 600 ± 30 °C. The temperature data reported by the SSQ-CCS align closely with the conventional thermocouple data, demonstrating consistency during both regular operations and the operational shift on day 2. Figure 11c presents the temperature data from the SSQ-CCS from day 421 to day 431. The thermocouple temperature data become unavailable. The operation temperature at the boiler tube remains 600 ± 30 °C, which proves the reliability of the SSQ-CCS for high-temperature sensing in harsh environments. 

Due to the unavailability of the operation parameters in the field testing, a direct comparison between the simulated temperature distribution on the boiler tubes and the temperature monitored by SSQ-CCS during the field testing is not feasible. In fact, our model setup leads to a higher temperature predicted than that monitored during the normal operations. Despite this, the predicted stress field under elevated temperature conditions remains lower than the yield stress of SS-347-H. This indirect validation proves the reliability of the proposed SSQ-CCS in high-temperature conditions.

## 4. Conclusions

We established a four-stage full-scale 3D multi-physics computational framework to assist the design, installation, and evaluation of the working conditions of a low-cost stainless-steel and quartz coaxial cable sensor (SSQ-CCS) [28] for distributed temperature monitoring of tubes in an industrial-scale coal-fired boiler. The ideal locations on the boiler tube panels for SSQ-CCS installation are identified as (1) away from the side walls, (2) closer to the penthouse floor of the boiler, and (3) not directly facing the flue gas flow. We investigate various operating conditions to analyze the temperature distribution and variation on the boiler tube. The boiler tube temperature has a linear response to the steam temperature and flue gas temperature, as well as a nonlinear response to the MFR of the steam. The correlation between the boiler tube temperature and SSQ-CCS temperature is established. Under the investigated conditions, the temperature variation along any cross-section of the boiler tube is less than 20 °C. The protection tube serves its role to protect the SSQ-CCS from direct contact with the flue gas. It also ensures temperature consistency between the boiler tube and the SSQ-CCS at the installed locations. The installation of the SSQ-CCS will lead to a temperature increase in the attached boiler tube with a magnitude of less than 20 °C. Through the structural thermal analysis, we ensure the reliability of the SSQ-CCS by predicting the stress distribution on the boiler tube due to the sensor installation. This study facilitates the successful field testing of the SSQ-CCS developed within this project [28]. This computational framework has the capability for future implementation in a variety of types of power plants. The integration of SSQ-CCS’s distributed temperature-sensing capability with systematic prediction and monitoring through computational modeling offers the feasibility to improve the current conditional-based monitoring technology with in situ distributed monitoring capability. Its potential economic contribution is significant. 

## Figures and Tables

**Table 1:** Material properties in the stage (C) and stage (D) models.

Material	Thermal Conductivity (W/(m·K))	Specific Heat (J/(kg·K))	Density (kg/m^3^)	Young’s Modulus (GPa)	Poisson’s Ratio	Thermal Expansion Coefficient (/K)
glass	1.4	670	2200	72	0.17	5.55 × 10^−7^
SS-347-H	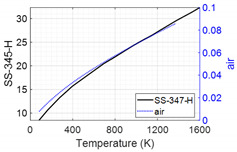	500	8030	195	0.27	1.2 × 10^−5^
air		1006.4	1.225			

## Data Availability

Data underlying the results presented in this paper are not publicly available at this time but may be obtained from the corresponding author upon reasonable request.
